# Prevalence and determinants of mental distress among university students in Ethiopia: a systematic review protocol

**DOI:** 10.1186/s13643-019-0966-z

**Published:** 2019-02-07

**Authors:** Berihun Assefa Dachew, Berhanu Boru Bifftu, Bewket Tadesse Tiruneh, Degefaye Zelalem Anlay, Meseret Adugna Wassie, Kim Betts

**Affiliations:** 1 Department of Epidemiology and Biostatistics, Institute of Public Health, College of Medicine and Health Sciences, University of Gondar, Gondar, Ethiopia; 2 School of Nursing, College of Medicine and Health Sciences, University of Gondar, Gondar, Ethiopia; 3 Department of Health Informatics, Teda Health Science College, Gondar, Ethiopia; 4 School of Public Health, Curtin University, Perth, Australia

**Keywords:** Prevalence, Mental distress, University students, Ethiopia

## Abstract

**Background:**

Mental distress is an important public health problem and becoming a common health problem among university students. This systematic review and meta-analysis will provide the pooled prevalence of mental distress and identify determinant factors associated with mental distress among university students in Ethiopia.

**Method:**

A systematic search of PubMed, EMBASE, and PsycINFO databases will be conducted. In addition, we will search grey literature resources such as a database/website of dissertations and theses, WHO websites, and websites of professional bodies. Reference lists of the selected articles will also be searched for additional articles. All observational studies reporting the prevalence of mental distress and/or associated factors among university students in Ethiopia will be included. Pooled prevalence with 95% confidence interval (95% CI) will be calculated using random-effects and quality-effects models. Subgroup and sensitivity analyses will be performed. Heterogeneity between studies and evidence of publication bias will also be assessed.

**Discussion:**

The proposed systematic review and meta-analysis will provide a pooled prevalence of mental distress and associated factors to assist policy-makers and programme managers in developing evidence-based mental health promotion and prevention programmes in university/college settings.

**Systematic review registration:**

PROSPERO CRD42017067223

**Electronic supplementary material:**

The online version of this article (10.1186/s13643-019-0966-z) contains supplementary material, which is available to authorized users.

## Background

Mental distress is a mental health problem characterized with a range of depressive, anxiety, or somatic symptoms such as headache, backache, and sleep problems [1, 2]. A person with mental distress may also present with confused emotions and hallucinations [2, 3]. The term is used when it has not been possible to obtain a specific diagnosis of mental disorder [3]. Mental distress affects a substantial proportion of the world population and is prevalent in universities worldwide [2, 4–9].

There are various factors that could contribute to mental distress among university students. The experience of new lifestyles, new roommates and friends, exposure to new cultures and environments, loss of traditional adult supervisors, and low social support are some of these factors [6, 10–12]. In addition, academic stressors, peer pressure, and financial distress have been recognized as risk factors for developing mental distress among students [5, 13]. However, many students do not seek help for their problems. Inability to manage these conditions early will lead to adverse impacts on their academic achievement [14, 15]. More importantly, mental distress is associated with an increased risk of substance use [16, 17] and suicidal behaviours [18–20].

In Ethiopia, mental and substance use disorders are common public health problems responsible for about 1897 disability-adjusted life years (DALYs) per 100,000 population [21]. Studies have shown that mental distress prevalence among university students in Ethiopia varies from 21.6 to 63.1% [5, 22–26]. Although several individual studies have reported the prevalence of mental distress and associated factors among university students in Ethiopia, to our knowledge there is no published systematic review and meta-analysis that shows pooled estimates of mental distress and its associated factors. Having a pooled prevalence of mental distress and identifying the associated factors would help policy-makers and programme managers in developing evidence-based mental health promotion and diseases prevention programmes. Therefore, the objective of this systematic review and meta-analysis is to review the existing literature, with the aim of quantifying the burden of mental distress and identifying factors associated with mental distress among university students in Ethiopia.

## Methods

This protocol adheres to the Preferred Reporting Items for Systematic Reviews and Meta-analysis Protocols (PRISMA-P) [27] (Additional file 1). The review protocol has been registered in the International Prospective Register of Systematic Reviews (PROSPERO) (ref CRD42017067223).

### Data sources and search strategies

A systematic search of PubMed (from its inception to present), EMBASE (1947 to present), and PsycINFO (1967 to present) databases will be conducted in accordance with a detailed search strategy. The search comprises both Medical Subject Headings (MeSH) and free text words (title and abstract word searches). We will use the following search terms: “Mental Disorders”[Mesh], “Somatoform Disorders/diagnosis”[Mesh]) in combination with “student*”, “university student*”, “college student*”, and “Ethiopian*”. The full electronic search strategies are included in the supplementary information (Additional file 2). In addition, we will search grey literature resources such as a database/website of dissertations and theses, WHO websites, and websites of professional bodies (e.g. mental health professional’s association and mental health charities). The reference lists of included studies will be manually searched for additional eligible articles. We will contact authors when additional information is required.

### Inclusion and exclusion criteria

All observational studies reporting the prevalence of mental distress and/or factors associated with mental distress among university students will be included. An article will be included if it meets the following criteria: (1) conducted solely or partly among university or college students, (2) reported the prevalence of mental distress using standardized instruments or questionnaires (such as Beck’s Depression Inventory, Patient Health Questionnaire-9, Self-reporting Questionnaire-20, or clinical interviews) or provided sufficient information to calculate prevalence, and (3) published in English. When studies include a sample partly composed of university/college students, we will extract required information to calculate prevalence. Conference abstracts, letters to editors, review, and commentary articles and studies where the participants are not human will be excluded. Non-English language studies will also be excluded as we do not have resources (to pay or find volunteers) to translate the data.

### Selection of studies for inclusion in the review

Titles and abstracts of studies retrieved from each database search will be stored and managed in an EndNote reference manager. Two review authors (BAD and MAW) will independently review the titles and abstracts of all studies and disagreements will be solved by discussion.

### Data extraction and management

Using a standardized data extraction form, two review authors (BAD and MAW) will independently extract data from eligible studies including first author’s last name, year of publication, study location, sample size, number of events, data on prevalence of mental distress, ascertainment of outcome, risk or protective factors examined in each study together with their respective odds ratio (OR) and 95% confidence intervals (CI), and information for assessment of the risk of bias.

### Quality assessment

Two review authors (BAD and MAW) will independently assess the quality of all included studies using the Newcastle-Ottawa Quality Assessment tool adapted for cross-sectional studies [28]. Discrepancies will be resolved by a third reviewer (BTT) if necessary. The tool takes into account the selection of participants, comparability, and assessment of outcome. For quantitative analysis, quality scores will be assigned by dividing each score by the score of the highest scoring study in the group.

### Data synthesis

The pooled prevalence (proportion) of mental distress and the pooled odds ratios (OR) of identified factors associated with mental distress with 95% CI will be calculated using random-effects [29] and quality-effects models [30]. Quality-effects meta-analysis will be used to examine how the quality of each study changed the pooled estimate compared with the results from random-effects meta-analysis. This analysis incorporates the quality score of each study in the calculation of the study weight, which is a robust and innovative technique to help minimize the estimator variance and account for subjectivity in quality assessment. Heterogeneity between the studies will be assessed using both Cochrane’s Q statistic and the *I*^2^ statistics. *I*^2^ value greater than 50% will be considered as indicative of substantial heterogeneity [31]. Funnel plots will be used to assess publication bias [32]. Double arcsine transformation will be used if there is variance instability.

Subgroup analyses will be performed to explore the sources of heterogeneity attributed to gender, sample size, year of publication, study quality, year of study, variation in thresholds, and assessment methods/instruments. Sensitivity analysis will be performed by excluding each study one by one and calculating a pooled estimate for the remainder of the studies. All statistical analyses will be performed using MetaXL version 5.3 and STATA14 Metaprop package.

### Presenting and reporting the results

A flow diagram will be included to outline the study selection process step by step, and a rationale provided for excluded studies. The characteristics and quality assessment of the included studies will be presented in tables. Pooled estimates will be presented using forest plots.

## Discussion

This will be the first systematic review and meta-analysis that will determine the pooled prevalence of mental distress among university students in Ethiopia. It will also identify factors associated with mental distress among students. By identifying the risk and protective factors of mental distress, the findings may help in developing evidence-based mental health promotion and diseases prevention programmes targeting university students.

### Potential limitations

It is anticipated that publication bias may pose as a limitation for this review. There is also a possibility of reporting and response set bias.

## Additional files

Additional file 1: PRISMA-P (Preferred Reporting Items for Systematic review and Meta-Analysis Protocols) 2015 checklist: recommended items to address in a systematic review protocol. (DOCX 15 kb)

Additional file 2: Example search used for identification of articles on the PubMed database. (DOCX 12 kb)

## Abbreviations

95% CI: 95% confidence interval
DALYs: Disability-adjusted life years
MeSH: Medical Subject Headings
PRISMA-P: Preferred Reporting Items for Systematic Reviews and Meta-analysis
WHO: World Health Organization
