# Supplementary material for: Prevalence and determinants of mental distress among university students in Ethiopia: a systematic review protocol
Source: Syst Rev. 2019 Feb 7;8:47. doi: 10.1186/s13643-019-0966-z (PMC6366100; doi:10.1186/s13643-019-0966-z)
Supplement: Supplementary file 1 — PRISMA-P (Preferred Reporting Items for Systematic review and Meta-Analysis Protocols) 2015 checklist: recommended items to address in a systematic review protocol. (DOCX 15 kb) [file 13643_2019_966_MOESM1_ESM.docx]

**Additional file 1:** PRISMA-P (Preferred Reporting Items for Systematic review and Meta-Analysis Protocols) 2015 checklist: recommended items to address in a systematic review protocol

| Section and topic | Item number | Checklist item | Reported on page # |
| --- | --- | --- | --- |
| **Administrative information** | | | |
| Title |  |  |  |
| Identification | 1a | Identify the report as a protocol of a systematic review | 1 |
| Update | 1b | If the protocol is as for an update of a previous systematic review, identify as such | NA |
| Registration | 2 | If registered, provide the name of the registry (such as PROSPERO ) and registration number | _ |
| Authors |  |  |  |
| Contacts | 3a | Provide name, institutional affiliation ,e-mail address of all protocol authors; provide physical mailing address of corresponding author | 1 |
| Contributions | 3b | Describe contributions of protocol authors and identify the guarantor of the review | 7 |
| Amendments | 4 | If the protocol represents an amendment of a previously completed or publish protocol, identify as such and list changes; otherwise, state plan for documenting important protocol amendments | NA |
| Support |  |  |  |
| Sources | 5a | Indicate source of financial or other support for the review | 7 |
| Sponsors | 5b | Provide name for the review funder and/or sponsor | NA |
| Role of sponsor or funder | 5c | Describe roles of funder(s),sponsor(s), and/or institution(s), if any , in developing the protocol | NA |
| **Introduction** | | | |
| Rationale | 6 | Describe the rationale for the review in the context of what is already known | 3 |
| Objectives | 7 | Provide an explicit statement of the question(s) the review will address with reference participants, interventions comparators, and outcomes (PICO) | 3 |
| **Methods** | | | |
| Eligibility criteria | 8 | Specify the study characteristics (such as PICO, study design, setting, time frame) and report characteristics (such as year considered , language, publication status) to be used as criteria for eligibility for the review | 4-5 |
| Information sources | 9 | Describe all intended information source (such as electronic database, contact with study authors, trial registers or other grey literature sources) with planned dates of coverage | 4 |
| Search strategy | 10 | Present draft of search strategy to be used for at list one electronic database , including planned limits such that it could be repeated | 4 |
| Study records: |  |  |  |
| Data management | 11a | Describe the mechanism(s) that will be used to manage records and data throughout the review | 4 |
| Selection process | 11b | State the process that will be used for selecting studies (such as two independent reviewers) through each phase of the review (that is screening, eligibility, and inclusion in meta-analysis) | 4 |
| Data collection process | 11c | Describe planned method of extracting data from reports ( such as piloting forms, done independently, in duplicate) , any process for obtaining and confirming data for investigators | 4-5 |
| Data items | 12 | List and define all variables for which data will be sought ( such as PICO item, funding sources), any pre -planned data assumptions and simplifications | 4 |
| Outcome and prioritizations | 13 | List and define all outcomes for which data will be sought , including prioritization of main and additional out comes, with rationale | 4 |
| Risk of bias in individual studies | 14 | Describe anticipated methods for assessing risk of bias of individual studies including whether this will be done at the outcome or study level ,or both ; state how this information will be used in data synthesis | 5 |
| Data synthesis | 15a | Describe criteria under which study data will be quantitatively synthesized | 5 |
|  | 15b | If data are appropriate for quantitative synthesis , describe planned summery measures, method of handling data and method of combining data from the studies, including any planned exploration of consistency (such as I^2^ , kendall’s Ʈ) | 5 |
|  | 15c | Describe any proposed additional analyses (such as sensitivity or sub group analyses, meta regression) | 5 |
|  | 15d | If quantitative synthesis is not appropriate, describe the type of summery planned | NA |
| Meat –bias (es) | 16 | Specify any planned assessment of meta –bias (es) (such as publication bias across studies , selective reporting within the studies) | 5 |
| Confidence in commutative evidence | 17 | Describe how the strength of the body of evidence will be assessed (such as GRADE) | 5 |

NA**:** Not applicable
