# Supplementary material for: Prevalence and determinants of mental distress among university students in Ethiopia: a systematic review protocol
Source: Syst Rev. 2019 Feb 7;8:47. doi: 10.1186/s13643-019-0966-z (PMC6366100; doi:10.1186/s13643-019-0966-z)
Supplement: Supplementary file 2 — Example search used for identification of articles on the PubMed database. (DOCX 12 kb) [file 13643_2019_966_MOESM2_ESM.docx]

**Additional file 2:** Example search used for identification of articles on PubMed database

**Search 1:** ("Mental Disorders"[Mesh]) OR "Somatoform Disorders "[Mesh] Filters: Humans; English

**Search 2:** ((student*) OR college student*) OR university student*

**Search 3:** Ethiopian*

**Search 4:** Search 1 AND Search 2 AND Search 3

Likewise, EMBASE, and PsycINFO databases will be searched using similar search terms tailored to each database
